# Pilot implementation study of a web-based men’s health screening app in primary care during COVID-19: a mixed-methods approach

**DOI:** 10.1186/s12913-024-11702-9

**Published:** 2024-10-11

**Authors:** Chor Yau Ooi, Chirk Jenn Ng, Anne Sales, Chin Hai Teo

**Affiliations:** 1 Department of Primary Care Medicine, Faculty of Medicine, Universiti Malaya, Kuala Lumpur, Malaysia; 2 Department of Family Medicine, Faculty of Medicine and Health Sciences, Universiti Malaysia Sarawak, Sarawak, Malaysia; 3 Health Services and Systems Research, Duke NUS Medical School, Singapore, Singapore; 4 Department of Family and Community Medicine, MU School of Medicine, University of Missouri, Missouri, USA

**Keywords:** Implementation, Web-based application, Screening, Primary care, COVID-19

## Abstract

**Background:**

The traditional delivery of healthcare services, including crucial preventive measures such as health screenings, faced significant disruption due to the COVID-19 pandemic. In response, eHealth technology emerged as a practical alternative for conducting screening services. This pilot study introduces ScreenMen, a web-based app for men’s health screening, implemented in a primary care setting. The study aims to assess patient uptake and healthcare provider’s acceptability and feasibility of implementing ScreenMen, emphasizing the importance of implementation science research in healthcare innovation.

**Methods:**

This study employed a mixed-method explanatory sequential design, using a tailored implementation intervention to implement ScreenMen in an urban health clinic. Quantitative phase focused on patient uptake of ScreenMen and healthcare provider involvement, utilizing Google Analytics and provider questionnaires. Qualitative phase, using in-depth interviews with providers, explored factors influencing uptake and implementation. Data analysis employed means and percentages for quantitative data and framework analysis for qualitative data.

**Results:**

We invited 47 healthcare providers to attend the ScreenMen implementation workshop, with 26 participating, resulting in a 55.3% participation rate. Throughout the five-month study, there were 75 recorded accesses, with a completion rate of 20%. The primary way users accessed the app was through QR codes on buntings (38.7%), followed by postcards (12%). In qualitative interviews with three healthcare providers, it was found that the Identify and prepare champions strategy was helpful, as these champions led the implementation and encouraged other providers to promote ScreenMen. The use of QR codes on buntings, part of the Provide education and training strategy, was effective due to their visibility in patient waiting areas. However, the Mandate change strategy was considered ineffective, as providers felt obligated rather than motivated to implement ScreenMen.

**Conclusion:**

This study highlighted the uptake of ScreenMen and found barriers and facilitators during the pilot implementation. Two useful strategies were Identify and prepare champions and QR codes while Mandate change was not helpful. Further studies are needed to study the effectiveness of these implementation strategies to implement web-based apps.

**Trial registration:**

Clinical Trial Number: NCT06388473 (Retrospectively registered 05/04/2024).

**Supplementary Information:**

The online version contains supplementary material available at 10.1186/s12913-024-11702-9.

## Background

The global impact of the COVID-19 pandemic was swift and far-reaching. Originating in Wuhan, China, the virus rapidly spread worldwide, causing disruptions across various healthcare services. Both hospital-based and primary-based healthcare services were affected to varying degrees. Essential preventive care, including health screenings, suffered disruptions during the pandemic as it was classified as non-essential. This is concerning as delayed detection of conditions such as cancer and non-communicable diseases (NCDs) can lead to severe complications. Moreover, COVID-19 demonstrated higher mortality rates in patients with uncontrolled NCDs like hypertension and type 2 diabetes [1]. A study reporting on health screening services showed screening of cervical cancer decreased by 7.5%, colorectal cancer decreased by 8.1%, and type 2 diabetes decreased by 4.5% [2].

Recognizing the need for an alternative approach to delivering health screening services, there is a suggestion to shift from traditional methods to utilizing eHealth technology. A scoping review revealed an increased usage of eHealth technology during the pandemic, encompassing telemedicine through platforms like Zoom, communication through emails and messaging systems like WhatsApp, and web-based applications [3, 4]. Notably, web-based applications have proven effective in delivering various health services and changing health behaviours [5, 6].

Men generally have lower life expectancy and higher mortality rates compared to women, largely due to health behaviours and societal norms associated with masculinity [7]. Men are more likely to engage in risky behaviours like smoking and excessive alcohol consumption and are less likely to participate in health-promoting activities such as regular screenings or self-examinations [8–10]. This reluctance to seek help is often linked to traditional masculine norms emphasizing independence, stoicism, and avoidance of activities perceived as feminine [11]. Masculinity affects health-seeking behaviours and can lead to denial of illness, reluctance to use healthcare services, and avoidance of preventive measures [12, 13]. However, this concept of masculinity is dynamic and can vary by age, socioeconomic status, and cultural context [11, 14, 15]. Recent studies suggest that positive masculinity attributes, such as taking responsibility and caring for one’s family, can promote better health practices among men [14, 15]. Thus, addressing men’s health issues may benefit from a positive approach that highlights their strengths and encourages healthy behaviours.

In Malaysia, men’s health screening programmes are offered at government health clinics, but a national study highlighted several barriers to their implementation [16]. The screening tool was considered overly lengthy and complicated, making it difficult to use efficiently, and many men were reluctant to take part. Furthermore, the clinics faced challenges such as insufficient staff, limited time, and a lack of resources, hindering the programme’s success [16]. As a result, a more effective tool is needed for implementing the men’s health screening programme. ScreenMen, a novel web-based app for screening in men was developed to educate and encourage men to engage in screening. The development of ScreenMen carefully considered various theories, evidence, and user requirements. The supporting evidence for ScreenMen was derived from recommendations provided by the United States Preventive Services Task Force (USPSTF) and the Malaysian Consensus Guide to Adult Health Screening for General Population Attending Primary Care Clinics [17–19]. Furthermore, the ScreenMen development team acknowledged the significance of incorporating a gender-sensitive approach to health interventions, which was reflected in the development process [20]. ScreenMen utilized a strength-based approach to promote health screening among men, drawing from masculinity concepts and leveraging male-friendly themes [21, 22]. Key elements of the app included: (1) Masculinity-Based Motivation: Emphasizing health to support and care for family, appealing to men’s roles as providers and protectors [23, 24], (2) Car Maintenance Analogy: Using concepts like the “Man MOT” and “Pit Stop Health Check” to make health screening relatable and engaging [25], (3) Dr. ScreenMen Avatar: A friendly, Superman-like figure to make the app formal and more appealing to men [26, 27], (4) Gender-Sensitive Design: Incorporating feedback from men and focusing on a male-only setting, although some users questioned the need for this gender-specific approach [28], (5) Online Platform: Utilizing web-based app to create a male-friendly virtual space for health advice and screening [29] and (6) Artificial intelligence: ScreenMen’s strengths include its use of artificial intelligence for tailored health advice and its continuous availability.

**Fig. 1:**
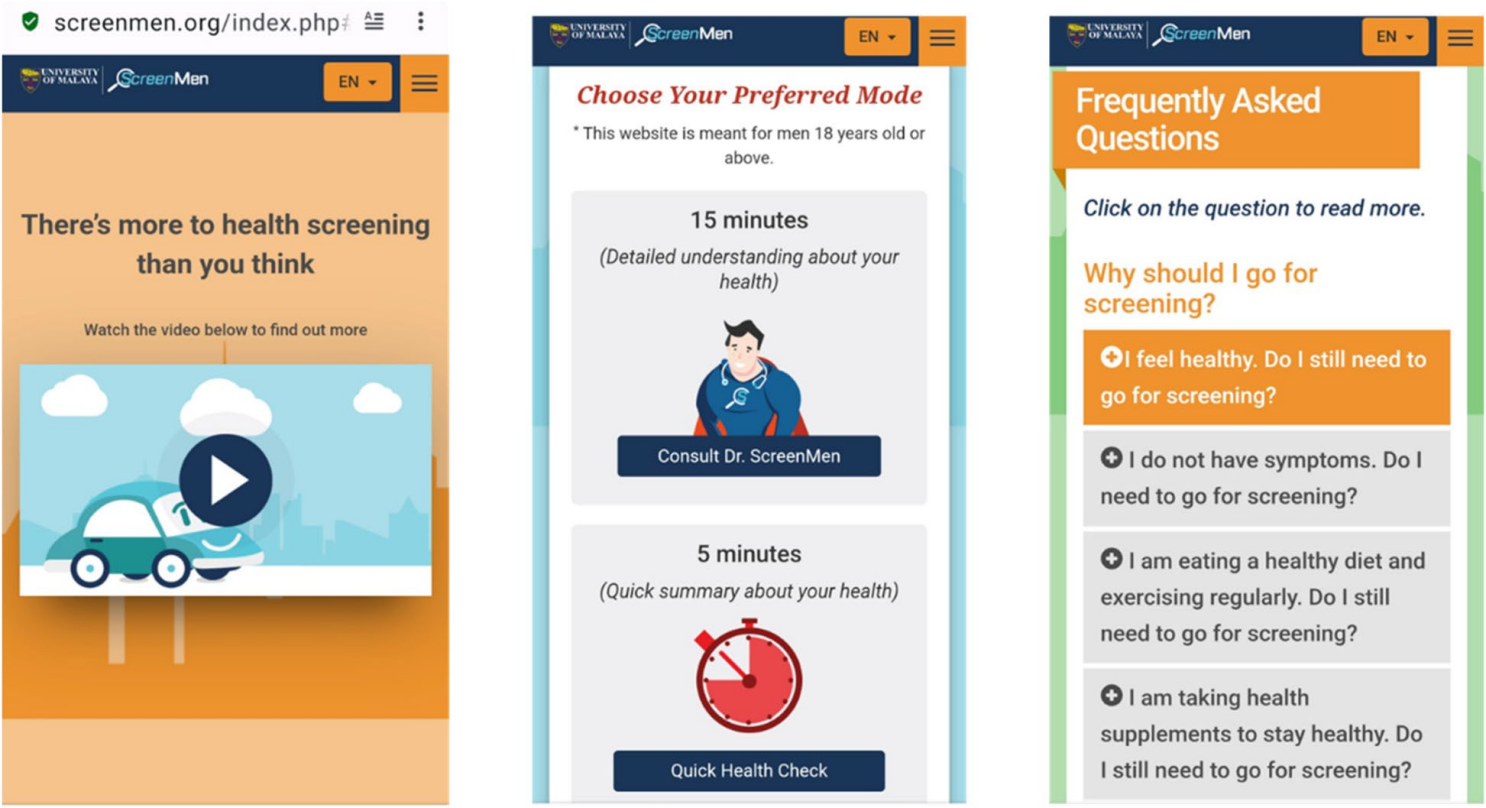
Screenshots of ScreenMen from a mobile device

ScreenMen consists of three key features:

1. What is Screening? This section includes an educational video addressing misconceptions about health screening. It emphasizes four key messages: Screen Now, Screen Regularly, Screen Despite Being Young and Healthy, and Screen Appropriately.
2. Check My Health: An interactive section where users can assess their health risks for 15 conditions, including obesity, unhealthy diet, and various diseases. The platform provides personalized, evidence-based health advice, replicating a real-life clinical consultation. Users receive a personalized health report with logistical information on where and when to screen and estimated costs.
3. Frequently Asked Questions: This section compiles commonly asked questions about health screening, addressing barriers and facilitators. ScreenMen is available in English, Malay, and Mandarin languages at https://screenmen.org [20]. Figure 1 shows some screenshots of ScreenMen.

Primary care is an optimal setting for the implementation of men’s health screening programmes because it serves as the initial point of contact for the general population regarding healthcare and plays a vital role as a “gatekeeper” for hospital care [30]. Healthcare providers in primary care possess extensive knowledge of preventive care and can deliver comprehensive care to patients who may receive diagnoses through screening [30]. Screening needs to be part of primary care to ensure abnormal findings are promptly addressed. Embedding men’s health screenings within primary care reduces the risk of overlooking or not acting upon important results.

However, the effectiveness of innovations often lies in their successful implementation within healthcare settings. Literature highlights the importance of implementation research, defined as the study of strategies to adopt and integrate evidence-based health interventions and change practice patterns within specific settings [31]. Implementation research employs a systematic approach, utilizing theories and frameworks to design strategies that facilitate the uptake, implementation, and sustainability of innovations in healthcare settings [32, 33]. Our scoping review reported that there are few studies on the implementation of web-based apps for screening [34]. Therefore, it is imperative to study how to implement a web-based app for screening in men in a primary care setting. The objective of this study is to assess the uptake of ScreenMen by patients and evaluate the acceptability and feasibility of recommending the application of the tailored implementation intervention designed to implement ScreenMen on the part of providers.

## Methods

### Study design

This pilot study employed a mixed-method explanatory sequential design [35]. The quantitative phase of this study examined the uptake of ScreenMen by patients and the participation of healthcare providers in implementing ScreenMen, recommending that patients use it. The qualitative phase aimed to explore in-depth the factors affecting the uptake of ScreenMen by patients and the factors affecting healthcare providers in implementing ScreenMen in a clinical setting. The mixed methods integration was effected in two points of the study: (1) while creating the interview guide for the semi-structured interviews, the development process was guided by the findings of the quantitative phase and (2) during the interpretation of the quantitative and qualitative results. Integration in a mixed methods study refers to the deliberate process of connecting or blending both quantitative and qualitative aspects. This is the fundamental characteristic that sets mixed methods research apart from studies that only incorporate some quantitative and qualitative data [36]. This study was reported using the Standards for Reporting Implementation Studies (StaRI) reporting guidelines [37].

### Setting

This study was conducted in a government health clinic, located in Kuala Lumpur, the capital city of Malaysia. This clinic is located in an urban setting and serves an estimated population of 100,000 people around its vicinity with a mean household income of RM 13,000 (USD 2,700). It is led by a family medicine specialist. The daily attendance of the clinic was estimated at 250 patients a day. The waiting time for the patients ranged from 5 to 30 min. Wi-Fi is not available for the public in the clinic.

### Participants

The participants were divided into two groups: healthcare providers and patients. Healthcare providers that were involved in the clinic health screening programme were included and the exclusion criteria were temporary healthcare providers (working in the clinic less than six months during the study period) and those who were away on leave. Patients who were aged 18 years and above were eligible to participate. For patients, women were included in the study as they can promote the app to men around them. Patients who did not understand Bahasa Malaysia, English or Mandarin, cognitively impaired, had an active psychiatric illness or were too ill were excluded.

### Tailored implementation intervention

For this study, a tailored implementation intervention was developed to implement ScreenMen. The tailored implementation intervention comprised of six implementation strategies: (1) Involve executive boards, (2) Mandate change, (3) Provide education and training, (4) Identify and prepare champions, (5) The use of information and communication technology and (6) Audit and provide feedback. The development of the tailored implementation intervention started with a study to identify the barriers and facilitators to implement ScreenMen in three government health clinics. Subsequently, a brainstorming session was held with a panel of experts to address the barriers and facilitators [38]. The solutions generated from the brainstorming session were mapped to the Expert Recommendations for Implementing Change (ERIC) implementation strategies taxonomy [33]. The implementation strategies that were selected were based on the appropriateness and feasibility to implement ScreenMen in a government health clinic setting. A detailed explanation on how the tailored implementation intervention was implemented is provided in Additional file 1 using the recommendations by Proctor et al. [39].

### Data collection

Quantitative data collection was divided among health care providers and patients. Eligible healthcare providers were invited to join the workshop for implementing ScreenMen. Before the workshop began, the participant information sheet and consent were given to the participants. After giving consent, they completed a background information questionnaire.

For patients, the data was collected using Google Analytics. Google Analytics is a publicly available tool that provides free data on website usage. Previous studies have used Google Analytics to evaluate the usage of websites that provide information on topics such as sexual health, genetics education, and antibiotic use, as well as those related to smoking cessation, knowledge translation, and osteoporosis. Therefore, Google Analytics supports analysis of user behaviour and developing strategies to enhance adherence [40]. For this study, Google Analytics was used primarily to analyse the usage of ScreenMen and where it was accessed from. It recorded the Internet Protocol address of users to identify every unique user and to avoid duplicates. It also captured the location from which the app was accessed. Thus, access to ScreenMen outside the vicinity of the clinic and Kuala Lumpur was excluded as it may not be part of the study. Google Analytics could analyse the quantity of pages visited by each user to determine if they successfully navigated through the app from start to finish. Patients were able to access ScreenMen through Quick Response (QR) codes or Uniform Resource Locator (URL) available on the buntings, posters and postcards that were available in the clinic and the clinic’s Facebook page. A unique QR code was assigned to each of the buntings, posters, postcards and Facebook page to identify from which modality the patients accessed ScreenMen. After the quantitative data collection and analysis was completed, the results were used to guide the development of the interview guide for the qualitative study.

Semi-structured, in-depth interviews were conducted with healthcare providers to collect qualitative data using an interview guide. The interview guide was divided into two parts. The first part explored the clinic situation during the COVID-19 pandemic and how it affected daily practice. The second part explored the pilot implementation of ScreenMen based on the RE-AIM (Reach, Effectiveness, Adoption, Implementation, Maintenance) framework [41] and data from the quantitative study. The RE-AIM framework, developed over two decades ago, is widely used in implementation outcome studies to address challenges in applying scientific findings to practice and policy [42]. It emphasizes internal and external validity, encouraging transparent reporting across its dimensions. Initially designed to improve research evaluation and reporting, it has been cited in over 700 publications and successfully applied in various settings, including clinical, community, and corporate environments, as well as policy initiatives [42, 43]. The interview guide was created by a team including the researcher (CYO), an implementation science expert (AES), and a family physician consultant (CJN). It included clear, open-ended questions centred on participants and structured around key themes (RE-AIM framework), with follow-up prompts for clarification. Flexibility was prioritized to encourage a natural flow of conversation, using question formats designed to elicit detailed responses. Ethical considerations were carefully addressed to prevent any harm [44]. The interview guide is available in the Additional file 2. The interview was conducted via the Zoom application online by a researcher (CYO). This method was used because of restrictions on physical movement during the pandemic. Studies have shown that the Zoom application is a viable method to collect data for qualitative study [45]. The interview sessions were recorded using the recording function in the Zoom application. All information collected during the research was kept strictly confidential. Saturation was achieved when there were no new themes after three interviews were conducted.

### Study outcomes

The study outcomes were guided by the RE-AIM framework. For this study, only Reach, Adoption, Implementation and Maintenance outcomes were measured. Table 1 shows the definition for each outcome based on the RE-AIM framework.

**Table 1:** Definition for each outcome based on the RE-AIM framework

RE-AIM dimensions	Healthcare providers		Patients
	Quantitative	Qualitative	Quantitative
**Reach**			
• Healthcare providers who participated in the ScreenMen implementation study. • Patients who accessed ScreenMen. • Patients who completed the ScreenMen app.	Number of healthcare providers who attended the workshop/Number of healthcare providers who were invited to the workshop.	• Factors affecting the participation of healthcare providers in the training workshop. • Factors influencing the usage of ScreenMen by patients.	• The number of patients who accessed ScreenMen. • The number of patients who completed the ScreenMen app.
**Adoption**			
Healthcare providers’ view on the implementation of ScreenMen.		• Factors affecting the participation of healthcare providers in adopting ScreenMen in daily practice. • Factors affecting the completion of ScreenMen by patients	
**Implementation**			
Healthcare providers’ view on the implementation strategies for ScreenMen.	The number of patients who accessed to ScreenMen from different modalities (buntings, posters, postcards, clinic Facebook page or keying directly the URL of ScreenMen).	Factors affecting the implementation of ScreenMen in daily practice.	
**Maintenance**			
Healthcare providers’ intention to continue to implement ScreenMen for the long term.		Intention to implement ScreenMen and factors influencing the decision.	

### Analysis

For the quantitative analysis, the data for the healthcare providers were entered into SPSS version 21. The data were analysed descriptively in means and percentages. The data from Google Analytics were analysed descriptively using a Microsoft Excel worksheet. Descriptive statistics performed were frequencies for the outcomes measured.

For the qualitative data, the Zoom recordings were transcribed verbatim, and the data was managed using NVivo version 10. The data was analysed using the framework analysis approach [46]. The researcher (CYO) familiarized himself with the data by rereading the transcripts and reviewing the Zoom recording. Next, the codes were aligned with the RE-AIM framework, with any codes that did not align being subject to open coding [47]. Themes were mapped onto the domains of the RE-AIM framework according to the relevance of the themes to the specific domain.

The researcher (CYO) took his reflexivity into account that a tailored implementation intervention was created for a clinic, addressing specific barriers and utilizing evidence-supported strategies. The researcher, involved in the entire process, aimed for positive feedback on implementing ScreenMen. As the COVID-19 pandemic was ongoing during the study, it was acknowledged that the clinic faced some challenges in implementing ScreenMen. However, it was recognized that not all the obstacles were solely due to the pandemic. In order to maintain objectivity during the interviews, efforts were made to impartially identify and clarify any limitations. Additionally, the data was interpreted objectively, without presuming that all limitations were caused by the COVID-19 pandemic. In this study, the researcher ensured rigor through credibility, dependability, confirmability, and transferability. Credibility was maintained by transparent communication with participants and avoiding personal biases in data analysis. Dependability was ensured through reflexivity and discussions with supervisors (CJN and AES). Transferability was achieved by providing a detailed description of the study process and findings, making the results relevant to others. Ethical approval was obtained from the National Medical Research Register of Malaysia (NMRR-20-2188-56086 [IIR]).

## Results

### The impact of the COVID-19 pandemic

Qualitative interviews were conducted with two physicians and one nurse. For the two physicians, one served as the clinic’s head and drove the ScreenMen implementation, and the other served as the overall implementation champion for the clinic. The nurse was the implementation champion for the nursing section.

The COVID-19 pandemic was a stressful time for healthcare providers. They were stressed and fatigued because of the extra workload and risk of contracting COVID-19 themselves. The healthcare providers were also worried they might spread the infection to their family members.

*“So*,* when a patient comes in*,* I will feel stress. Because we are at risk every time (of COVID-19 infection).” (SN1)*.

*“My main concern is bringing back the disease to my family members. Personally*,* on a very personal level*,* I*,* although I’m living with my family under the same roof*,* I am keeping a distance from them.” (Dr1)*.

*“In my opinion*,* when considering whether Covid has an effect*,* I believe it certainly does. Everyone seems fatigued and perhaps even overworked.” (Dr2)*.

As for the clinic, shortages of healthcare providers to run the clinic was a challenge as manpower was deployed for COVID-19-related works.

*“Busy not because of our clinical work but apart from our clinical work we have our Covid-related job. Well*,* as you’re aware*,* we’re responsible for deploying our personnel*,* including myself as well as our medical officer*,* each with specific roles and duties at the Covid Assessment Centre.” (Dr2)*.

*“…because in late February we started our planning for vaccination for the healthcare workers. We initiate the setup*,* organization*,* and preparation of the vaccination centre for the general public only in April.“(Dr2)*.

Regarding the patients receiving treatment at the clinic, the standard of care suffered due to the impact of the COVID-19 pandemic. This occurred because only individuals with urgent medical needs were permitted to access the clinic during this time.

*“I would say the quality of care somewhat would have to be compromised.” (Dr1)*.

*“So*,* we had to limit the number of patients that come to our clinic that time. Because we wanted to avoid a huge traffic flow. So instead of seeing them*,* for all those chronic patients*,* instead of seeing them on like a regular basis*,* we just renewed their medication and reviewed their blood results. If everything is normal*,* we will just arrange the next appointment for them.” (Dr1)*.

### The ScreenMen implementation outcomes based on RE-AIM framework

#### Reach

We approached 47 healthcare providers to join the ScreenMen implementation workshop and 26 of them participated (55.3%). The healthcare providers cited that the implementation helped raise awareness about men’s health but not all were interested in joining. Some healthcare providers were not willing to be involved as they felt they were busy enough.

*“I think our intention has been to raise awareness regarding men’s health in the community.” (Dr1)*.

*“Realistically speaking*,* not everyone would be interested to get involved in any programme*,* some people would just prefer to have a more laid-back lifestyle and not get themselves involved in too many programmes. So*,* by getting them involved right*,* how much*,* are they willing to go full*,* full-hearted out? That is the question to answer.” (Dr1)*.

**Fig. 2:**
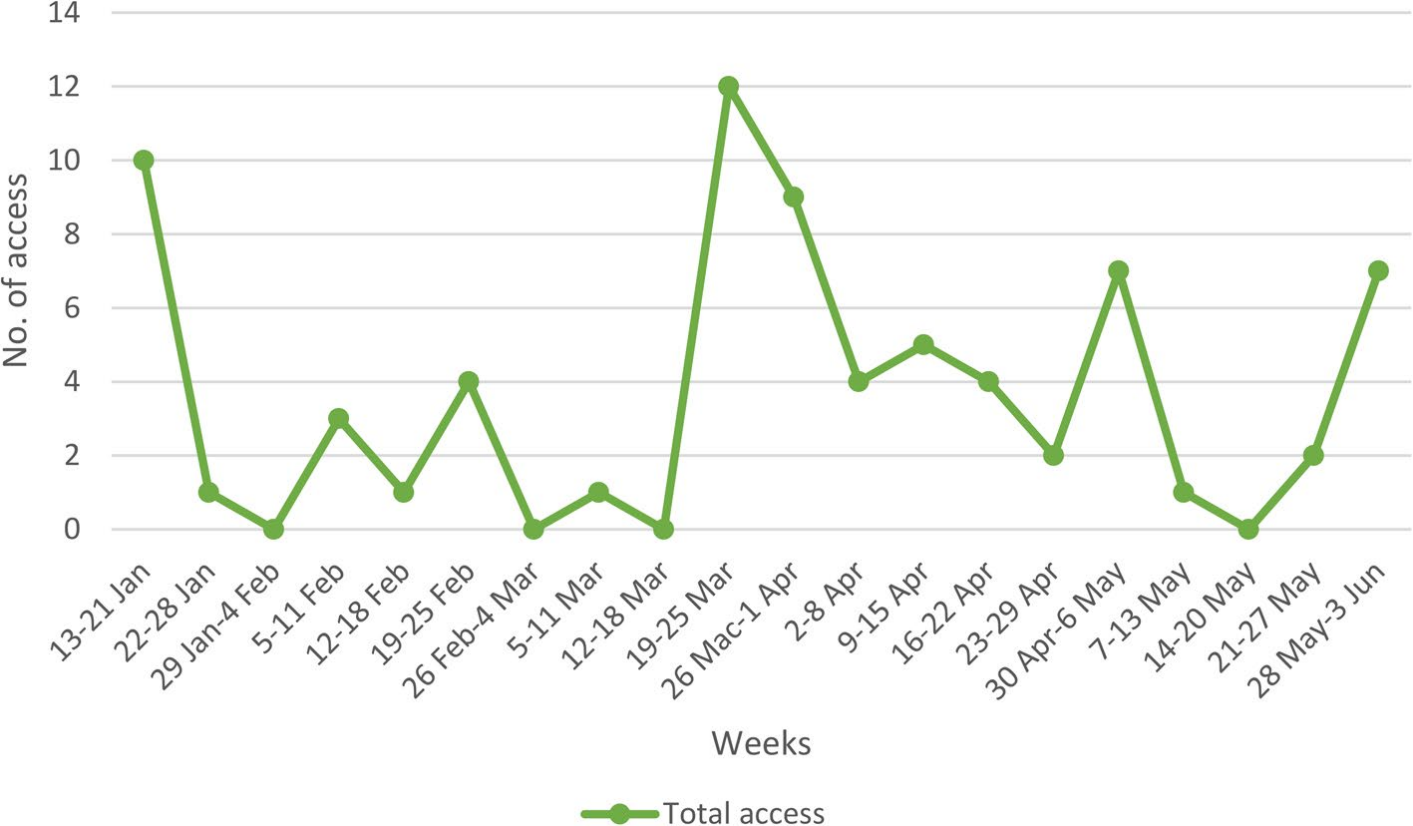
Weekly access to ScreenMen

A total of 75 accesses were recorded for the five months duration of the study. Figure 2. shows the trend of access to ScreenMen over time. The access was relatively high during the start of implementation but dropped in February and March. There was an increase in access to ScreenMen by the end of March with spikes in May and June.

In the qualitative analysis, healthcare providers observed that overall access to ScreenMen was lower than anticipated. They attributed this decline to the COVID-19 pandemic lockdown, which led to fewer patients visiting the clinic. However, by the end of March, access to ScreenMen had increased due to several factors. The vaccination program had commenced, bringing more patients to the clinic. Additionally, the ScreenMen champion actively encouraged patients to use the website during their visits. Furthermore, patients became more health-conscious as they prepared for their COVID-19 vaccinations, making them more inclined to use the app to check their health status.

*“Yes*,* because in fact in Jan*,* I think we got the MCO (lockdown) back then.” (SN1)*.

*“We began administering vaccinations*,* and consequently*,* patients started arriving with less fear. During that period*,* we actively promoted the Covid-19 vaccine*,* which led to patients inquiring about vaccination. As they asked about vaccination*,* we seized the opportunity to promote any health services in the clinic.” (SN1)*.

*“So*,* meaning that those people who clicked this website*,* they did it because I personally asked them to click it. So*,* they have to do it on the spot.” (Dr1)*.

*“When they (patients) wanted the COVID-19 vaccination*,* they were concerned about their self-health*,* whether they are suitable to get the vaccine or not.” (SN1)*.

Fifteen patients completed the ScreenMen app, giving a completion rate of 20%. From the qualitative analysis with healthcare providers, it was noted that they felt the overall completion rate was low. Several factors were identified as contributing to this: (1) The app was too lengthy, causing patients to lose interest in completing it, (2) A lack of incentives for patients to finish the app, (3) Concerns about privacy, and (4) Patients having difficulty understanding the questions in the app.

*“Maybe they lost patience.” (Dr1)*.

*“Maybe they couldn’t really understand the questions.” (Dr1)*.

*“I believe the incentive*,* what they stand to gain if*,* for instance*,* they undertake this action. However*,* despite our constant reminders*,* individuals may desire rewards beyond just maintaining their health. This suggests that certain individuals are primarily motivated by material possessions.” (Dr2)*.

*“When we tell them (patients) about apps on the phone*,* are web-based*,* so they were afraid that their personal details might be accessed by other people. They were really worried about this.” (SN1)*.

#### Adoption

The healthcare providers felt that the ScreenMen app was easy to use for the patients and is multilingual, therefore can cater for more people. However, barrier to adoption was busyness with clinic workload and lack of resources. Some of the healthcare providers were involved in multiple programmes.

*“I find the app is appealing due to its multilingual feature. Traditional paper-based screenings typically only cater to one language*,* Malay. Therefore*,* not everyone feels comfortable with just one language*,* especially the elderly or those with limited education. It’s important to provide options in languages they are familiar with.” (SN1)*.

*“On top of that*,* everybody already has their responsibility in the clinic. So it is not easy to juggle between*,* you know getting involved in a programme and at the same time you know needing to deal with the clinic work.” (Dr1)*.

*“Yes*,* the lack of manpower. I mean this programme (implementing ScreenMen) is pretty much*,* as far as this clinic is concerned*,* run by just a few people. I think it would be great if more people can get involved.” (Dr1)*.

#### Implementation

The majority of patients accessed the app using the QR code from the bunting (38.7%) followed by the postcards (12%). The healthcare providers felt that the bunting was useful to promote access to ScreenMen as it was large and placed at strategic locations around the clinic, namely the waiting areas. Postcards were found to be convenient to share with the patients about ScreenMen. However, healthcare providers also felt that patients who took the postcards would also forget about them and throw them away without accessing the app.

*“I think bunting because we put it in the patient waiting area. While they (patients) wait to see the doctor*,* they can access the app.” (SN1)*.

*“Bunting is bigger so people would just sit there and just scan.” (Dr2)*.

*“When considering postcards*,* I imagine we distribute them*,* and recipients take them home. Later*,* I anticipate they might misplace them and forget where they placed them. Occasionally*,* I notice postcards left on chairs in the waiting area*,* leading me to believe that many people will indeed forget about them.” (SN1)*.

Healthcare providers cited that having Implementation champions was important as they helped to lead and oversee the programme. Champions could also remind healthcare providers to promote the app to the patients. However, healthcare providers felt that Mandate change was not helpful and viewed this strategy as a double-edged sword that can either work or fail. By using Mandate change to ‘force’ healthcare providers to implement ScreenMen, they might do it but albeit reluctantly and this might compromise the quality of the implementation.

*“I think having a person in charge. I think a programme would not be able to go far without a person in charge*,* you need a leader to help to sort of oversee everything.” (Dr1)*.

*“I think is person-in-charge. So*,* he/she will be the one who helps remind us every time we need to do the ScreenMen and remind us about the app*,* to promote to the patient.” (SN1)*.

*“It operates as a double-edged sword*,* offering the advantage of garnering attention*,* but simultaneously presenting drawbacks. This duality is among its flaws because*,* in reality*,* not everyone is inclined to participate in programmes. Some individuals simply prefer a more relaxed lifestyle and avoid involvement in numerous initiatives. Thus*,* the extent of their commitment becomes a pertinent question. While enforcing the first strategy (mandate change) may compel participation*,* it risks compromising the quality of the work.” (Dr1)*.

#### Maintenance

The healthcare providers had the intention to continue using ScreenMen for the long term. The reasons that the healthcare providers would want to continue using ScreenMen were (1) timesaving, (2) user-friendly, and (3) helps healthcare providers to assess the patient.

*“Sure*,* why not. I see no harm in continuing as long as it (ScreenMen) doesn’t slow down the clinic operation.” (Dr1)*.

*“I believe in the application*,* and then I believe that it serves its purpose. I think it is very user friendly*,* especially for those who know how to use it and is I think*,* saves time also.” (Dr2)*.

*“So*,* I think the app helps the doctor to assess the patient because it will assess all the things already. So*,* the patient’s doctor can assess more in detail than to spend the time to find out what is the problem with the patient.” (SN1)*.

## Discussion

### Principal findings

This study highlighted the implementation of ScreenMen in a primary care clinic. As this was the first time ScreenMen, or any web-based screening approach, was implemented in a primary care clinic setting, this study provides useful insight into how it could be done.

For ‘Reach’ among providers, about half of the healthcare providers (55.3%) participated in the workshop. From the qualitative interviews, the barriers cited were that not all healthcare providers were interested in the programme, and they were not obliged to implement ScreenMen. According to a systematic review, the views of healthcare providers regarding eHealth technology usage were a significant factor, and a positive outlook was closely related to an increase in uptake [48].

As for ‘Adoption’ of ScreenMen, clinic workload and lack of resources were cited as barriers. These findings were consistent with the literature as increased workloads and lack of resources to implement eHealth technology were significant barriers [49, 50]. Lack of resources, in particular, was reported as a critical barrier to eHealth technology in developing countries [51]. Therefore, this barrier must be addressed before a full-scale implementation is feasible. A facilitator to ‘Adoption’ was that ScreenMen could be a replacement for the current paper-based screening method. The successful implementation of eHealth technology was found to be heavily dependent on the incorporation of technology that aligned with existing clinical practices and could be seamlessly integrated, as reported in a systematic review [48].

As for patients’ usage of ScreenMen, there was a discrepancy between the number of accesses compared to the completion rate. The completion rate for ScreenMen was relatively low compared to the number of accesses. There were a few reasons found in the qualitative interviews. One possible explanation from the qualitative study was the app was too lengthy. Two systematic reviews stated that the simplicity of using eHealth technology was a significant aspect of its acceptance [48, 49]. Although giving incentives to patients was a facilitator for using the app, the cost of doing so may not be viable in the long term. Regarding privacy, few studies have reported that confidentiality was an important factor in the usage of eHealth technology [49, 50]. Patients’ understanding of the questions in the app might be related to the eHealth literacy level of the patients. A study conducted locally reported that eHealth literacy among Malaysians was low and this might impact the usage of eHealth technology among patients [52]. Poor digital health literacy was the most cited barrier in a systematic review [50].

From the interviews, one of the reasons there was an increase in the uptake of ScreenMen was the patients accessed the app in the clinic on the spot. Another study also found that making patients complete the app in the clinic or with assistance from healthcare providers yielded a higher uptake. That study reported that 71% of patients completed the app in the clinic, compared to 30% when completed by the patients themselves [53]. However, in some settings, this method may not be feasible as most healthcare providers are busy with other clinical work and are not able to monitor if patients access the app. Patient empowerment and self-management may be the way forward as it was reported as a facilitator contributing to the success of eHealth technology [49].

One of the implementation strategies that was useful was the use of promotional materials to promote ScreenMen to the patients. Among the four modalities (poster, postcard, bunting, and Facebook), bunting had the highest number of accesses by patients. Previous studies have shown that health promotional materials (posters/leaflets) in the waiting room were noticed by patients [54, 55]. Patients found the information in the posters to be useful and wanted to know more after reading them. Although bunting was not mentioned specifically in the literature, it is similar to a poster but in a larger format. Therefore, it could be assumed that the function of bunting is similar to that of a poster. The usage of QR codes in these promotional materials enabled easier access to ScreenMen. As the buntings were located in strategic locations in the waiting room with high patient traffic, the patients can just scan the QR code, and thus have access to the app. QR codes have been used widely in health education for various purposes. A scoping review reported that QR codes were used to increase participant engagement, for simulation training, for just-in-time learning and to facilitate administrative tasks in training [56]. Another study looking into distributing patient information brochures using QR codes revealed that most patients found QR codes to be effortless to utilize and favoured them over printed leaflets. QR codes are a recognizable, efficient, and hands-free technology, thereby enhancing infection control protocols while reducing patient interaction with diverse surfaces and objects [57].

Another implementation strategy that was found to be useful was Implementation champions. Having champions taking charge of the implementation of ScreenMen helped to lead and oversee the programme. The literature found that champions may enhance the success of the implementation of eHealth technology [58]. Other systematic reviews on the effectiveness of champions in implementation were mixed [59, 60]. These findings were due to insufficient information on the champions’ attributes and training, as well as a failure to analyse the necessary combination of personal traits and training that would be required for the successful implementation of innovation [60]. For this study, the champions were briefed about their roles and responsibilities clearly regarding the implementation of ScreenMen. This might explain why healthcare providers found this strategy to be useful. However, more studies are needed for conclusive outcomes.

Mandate change was found to be unhelpful as an implementation strategy for ScreenMen. The healthcare providers felt that although Mandate change can facilitate implementation, the quality of the work may be compromised. Based on a systematic review, it was reported that the endorsement and active promotion of technology by senior professionals play a critical role in ensuring its success [48]. In another systematic review, to enhance the successful implementation of eHealth systems, it is beneficial to have leadership involvement throughout the development and implementation stages. Conversely, a lack of leadership can hinder the implementation process. Additionally, the support of management is a crucial factor in achieving implementation success [58]. The difference in perspective regarding Mandate change found in this study may be attributed to the way the implementation strategy was executed. As the clinic head only issued a memo to endorse the implementation of ScreenMen without follow-up action, this may not be enough to get a buy-in from the healthcare providers. In future studies, the execution of Mandate change may need to be explored further to enhance the implementation of the app.

### Impact of COVID-19

This pilot implementation study was conducted during the COVID-19 pandemic. According to interviews with healthcare providers, a significant number of them felt overwhelmed by the increased workload and anxiety about the disease. They were worried not only about their own risk of contracting the disease but also about potentially spreading it to their families. The quality of care of patients was also deemed to be compromised because of the limit of the patient load imposed by the clinic to reduce disease transmission. Most non-urgent services in the clinic were halted, which included health screening. It is established that patients with comorbidities, especially hypertension and diabetes, have a higher complication rate when infected with COVID-19 [1]. With health screening services disrupted during the pandemic, individuals with undiagnosed NCDs are at increased risk both of infection and of severe illness if they become infected. In low-income countries, as well as in some middle-income countries, the combination of undiagnosed NCDs and nutritional and other factors leading to poor health status significantly increases the risk of contracting COVID-19 and experiencing severe forms of the disease if contracted. Therefore, there was an urgency for more innovative and pragmatic approaches to implementing health screening during the pandemic. Technology can be used to overcome barriers to screening by improving accessibility, motivating and reminding individuals to get screened. Using ScreenMen as a screening tool for men reduced the need for clinic visits or face-to-face contact with healthcare providers to complete paper-based screening tools, and costs incurred travelling to and from the clinic. Individuals only need to attend a clinic for essential screening tests, procedures and consult with providers after that. This reduces waiting time and helps decongest the clinics. While modifications are essential for current screening methods, all these measures can be continued beyond the COVID-19 pandemic as an option for delivering community-based health screening. For instance, using a standardised, evidence-based, web-based tool may reduce screening practice variation and reduce waste by reducing unnecessary screening tests.

The COVID-19 pandemic has exposed many weaknesses in most healthcare systems globally. Fortunately, it also creates opportunities to change our practice for the better. Implementing ScreenMen during this trying time showed that not only it could be done, but it also offered an alternative screening method that may be beneficial for patients.

### Strengths and limitations

One of the strengths of this study was using the RE-AIM framework. Using the framework to guide the study provided a comprehensive means to evaluate the implementation of ScreenMen, both at the clinic and patient levels. Another strength of this study was the mixed-method study design. The mixed-method sequential explanatory design allowed a comprehensive understanding of the implementation process. In this study, integrating the quantitative and qualitative findings helped to explain some of the possible reasons for the usage of ScreenMen by patients, explaining the trend of access to ScreenMen and the low completion rate. This design also shed some insights into which implementation strategies were helpful to implement ScreenMen. However, there were several limitations. Participation in this study was limited. For patients, factors such as the lockdown, fear of visiting the clinic due to the risk of contracting COVID-19, and restrictions on the number of patients allowed in the clinic likely contributed to the low turnout. As for healthcare providers, their focus was primarily on managing the COVID-19 pandemic, especially during a surge in cases, making the implementation of ScreenMen a lower priority during that time. Although efforts were made to contact the healthcare providers through multiple phone calls and messages, the participation rate remained low. However, the three healthcare providers who participated in the study were most actively involved in the implementation of ScreenMen and therefore their insight was valuable. Another possible limitation was the interviews were only conducted with the healthcare providers and not the patients. Therefore, the views of the patients are not represented.

### Recommendations

The findings of this study can facilitate the implementation of a web-based screening app. The introduction of ScreenMen provided a viable solution, enabling screening to continue with minimal resource requirements. Patients were simply required to acquire the URL and conduct the screening themselves. Only those needing further testing had to visit the clinic. Consequently, this approach effectively reduced the number of unnecessary clinic visits, particularly amidst the pandemic. The tailored implementation strategies, designed specifically for Malaysia’s primary care setting, increase the chances of successful implementation, even beyond the pandemic. With limited research on the implementation of web-based screening tools, our findings contribute some novel insights on effective approaches. Despite attempting to address all challenges, resource limitations and the pandemic hindered a comprehensive resolution. The six proposed strategies can guide the implementation of similar apps. Key factors for implementation include early stakeholder engagement, staff training, appointing implementation champions, and distributing promotional materials with QR codes. Moving forward, addressing privacy concerns, simplifying the app, offering incentives, engaging patients, and providing essential resources, like Wi-Fi or kiosks in clinics, may further facilitate implementation.

## Conclusion

This study evaluated the implementation of ScreenMen in a government primary care clinic during the COVID-19 pandemic. Despite the limitations, this study was able to monitor and identify implementation strategies that facilitate the uptake of ScreenMen. Two useful strategies were clinical champions and QR codes while mandate change was not helpful. Subsequent research can focus on expanding the implementation of ScreenMen to more clinics and outside the clinical setting while considering the lessons derived from this pilot study. It is important to note that this study took place during the Covid-19 pandemic, and the implementation process may vary under non-pandemic circumstances. For full-scale evaluation of the effectiveness of the tailored implementation intervention, a hybrid trial type 2 or hybrid trial type 3 study design might be the way forward [61].

## Supplementary Information

Supplementary Material 1.

Supplementary Material 2.

## Data Availability

The datasets used and/or analysed during the current study are available from the corresponding author on reasonable request.

## Abbreviations

ERIC: Expert Recommendations for Implementing Change
NCDs: Non-communicable diseases
QR: Quick Response
RE-AIM: Reach, Effectiveness, Adoption, Implementation, Maintenance
StaRI: Standards for Reporting Implementation Studies
URL: Uniform Resource Locator
USPSTF: United States Preventive Services Task Force

## References

[1] Lu L, Zhong W, Bian Z, Li Z, Zhang K, Liang B, et al. A comparison of mortality-related risk factors of COVID-19, SARS, and MERS: a systematic review and meta-analysis. J Infect. 2020;81(4):e18–25. 10.1016/j.jinf.2020.07.002 PMC7334925 32634459

[2] Laing S, Johnston S. Estimated impact of COVID-19 on preventive care service delivery: an observational cohort study. BMC Health Serv Res. 2021;21(1):1107. 34656114 10.1186/s12913-021-07131-7 PMC8520349

[3] Abd-Alrazaq A, Hassan A, Abuelezz I, Ahmed A, Alzubaidi MS, Shah U, et al. Overview of technologies Implemented during the First Wave of the COVID-19 pandemic: scoping review. J Med Internet Res. 2021;23(9):e29136. 34406962 10.2196/29136 PMC8767979

[4] Murthy S, Kamath P, Godinho MA, Gudi N, Jacob A, John O. Digital health innovations for non-communicable disease management during the COVID-19 pandemic: a rapid scoping review. BMJ Innov. 2023;9(1):3–18.

[5] Wantland DJ, Portillo CJ, Holzemer WL, Slaughter R, McGhee EM. The effectiveness of web-based vs. non-web-based interventions: a meta-analysis of behavioral change outcomes. J Med Internet Res. 2004;6(4):e40. 15631964 10.2196/jmir.6.4.e40 PMC1550624

[6] Bailey JV, Murray E, Rait G, Mercer CH, Morris RW, Peacock R et al. Interactive computer-based interventions for sexual health promotion. Cochrane Database Syst Rev. 2010;8(9):Cd006483. 10.1002/14651858.CD006483.pub2 PMC13139710 20824850

[7] Tan HM, Ng CJ, Ho CCK, Teo CH. Asian men’s Health Report. Kuala Lumpur: Malaysia Men’s Health Initiative; 2013.

[8] WHO. Prevalence - adult age-standardized - Current smoking of any tobacco product. 2015 http://apps.who.int/gho/data/node.main.1250?lang=en. Accessed on 5 Sept 2024.

[9] WHO. Total Consumption of Alcohol. 2015 http://apps.who.int/gho/data/node.main.A1032?lang=en. Accessed on 5 Sept 2024.

[10] Dryden R, Williams B, McCowan C, Themessl-Huber M. What do we know about who does and does not attend general health checks? Findings from a narrative scoping review. BMC Public Health. 2012;12(1):723. 22938046 10.1186/1471-2458-12-723 PMC3491052

[11] Courtenay WH. Constructions of masculinity and their influence on men’s well-being: a theory of gender and health. Soc Sci Med (1982). 2000;50(10):1385–401. 10.1016/s0277-9536(99)00390-1 10741575

[12] Brown S. What makes men talk about Health? J Gend Stud. 2001;10(2):187–95.

[13] Fish JA, Prichard I, Ettridge K, Grunfeld EA, Wilson C. Psychosocial factors that influence men’s help-seeking for cancer symptoms: a systematic synthesis of mixed methods research. Psychooncology. 2015;24(10):1222–32. 26202128 10.1002/pon.3912

[14] Houle J, Meunier S, Coulombe S, Tremblay G, Gaboury I, De Montigny F, et al. Masculinity ideology among male workers and its relationship to self-reported health behaviors. Int J Men’s Health. 2015;14(2):163–82.

[15] Rochelle TL. Masculinity, health behavior, and age: an examination of Hong Kong Chinese men. Psychol Men Masc. 2015;16(3):294–303.

[16] Ng CJ, Teo CH, Ang KM, Kok YL, Ashraf K, Leong HL, et al. Barriers to implementing a national health screening program for men in Malaysia: an online survey of healthcare providers. Malays Fam Physician. 2020;15(1):6–14. 32284799 PMC7136681

[17] USPSTF, About. the USPSTF. 2021 https://www.uspreventiveservicestaskforce.org/uspstf/about-uspstf. Accessed on 5 Sept 2024.

[18] Tong SF, Krishnapillai ADAPS, Miskan M, Yasin MM, Keat NK, Ismail AH, et al. Malaysian Consensus Guide to Adult Health Screening for General Population Attending Primary Care clinics. First ed. Kuala Lumpur: Family Medicine Specialists Association of Malaysia; 2015. p. 113.

[19] Force USPST. USPSTF A and B recommendations. 2017 https://www.uspreventiveservicestaskforce.org/uspstf/recommendation-topics/uspstf-and-b-recommendations. Accessed on 5 Sept 2024.

[20] Teo CH, Ng CJ, Lo SK, Lim CD, White A. A Mobile web app to Improve Health Screening Uptake in men (ScreenMen): utility and usability evaluation study. JMIR mHealth uHealth. 2019;7(4):e10216. 30985280 10.2196/10216 PMC6487344

[21] MacDonald J. A different Framework for looking at men’s Health. Int J Mens Health. 2016;15(3):283.

[22] Robertson S, Williams R. Men, public health and health promotion: towards a critically structural and embodied understanding. In:, editors BGSR, editor. Men, masculinities and health: critical perspectives. New York: Palgrave Macmillan; 2010. pp. 48–66.

[23] Fazli Khalaf Z, Low WY, Ghorbani B, Merghati Khoei E. Perception of masculinity amongst young Malaysian men: a qualitative study of university students. BMC Public Health. 2013;13:1062. 24215138 10.1186/1471-2458-13-1062 PMC3840654

[24] Ng CJ, Tan HM, Low WY. What do Asian men consider as important masculinity attributes? Findings from the Asian men’s attitudes to life events and sexuality (MALES) study. J Men’s Health. 2008;5(4):350–5.

[25] Baker P. Man MOT’: a new approach to primary care for men. Trends Urol Men’s Health. 2017;8(1):13–6.

[26] Allen JD, Kennedy M, Wilson-Glover A, Gilligan TD. African-American men’s perceptions about prostate cancer: implications for designing educational interventions. Soc Sci Med (1982). 2007;64(11):2189–200. 10.1016/j.socscimed.2007.01.007 17399877

[27] Praptika Y, Putra GMN, editors. The Representation of Masculinity in South Korean Reality Show The Return of Superman. 2016.

[28] Robertson C, Archibald D, Avenell A, Douglas F, Hoddinott P, van Teijlingen E et al. Systematic reviews of and integrated report on the quantitative, qualitative and economic evidence base for the management of obesity in men. Health technology assessment (Winchester, England). 2014;18(35):v-vi, xxiii-xxix, 1-424. 10.3310/hta18350 PMC4781190 24857516

[29] Scholten MR, Kelders SM, Van Gemert-Pijnen JE. Self-guided web-based interventions: scoping review on user needs and the potential of Embodied Conversational agents to address them. J Med Internet Res. 2017;19(11):e383. 29146567 10.2196/jmir.7351 PMC5709656

[30] Emery JD, Shaw K, Williams B, Mazza D, Fallon-Ferguson J, Varlow M, Trevena LJ. The role of primary care in early detection and follow-up of cancer. Nat Rev Clin Oncol. 2014;11(1):38–48. 24247164 10.1038/nrclinonc.2013.212

[31] Glasgow RE, Vinson C, Chambers D, Khoury MJ, Kaplan RM, Hunter C. National Institutes of Health Approaches to dissemination and implementation science: current and future directions. Am J Public Health. 2012;102(7):1274–81. 22594758 10.2105/AJPH.2012.300755 PMC3478005

[32] Nilsen P. Making sense of implementation theories, models and frameworks. Implement Sci. 2015;10:53. 25895742 10.1186/s13012-015-0242-0 PMC4406164

[33] Powell BJ, Waltz TJ, Chinman MJ, Damschroder LJ, Smith JL, Matthieu MM, et al. A refined compilation of implementation strategies: results from the Expert recommendations for Implementing Change (ERIC) project. Implement Sci. 2015;10(1):21. 25889199 10.1186/s13012-015-0209-1 PMC4328074

[34] Ooi CY, Ng CJ, Sales AE, Lim HM. Implementation strategies for web-based apps for screening: scoping review. J Med Internet Res. 2020;22(7):e15591–e. 32706655 10.2196/15591 PMC7400029

[35] Schoonenboom J, Johnson RB. How to construct a mixed methods Research Design. Kolner Z fur Soziologie Und Sozialpsychologie. 2017;69(Suppl 2):107–31. 10.1007/s11577-017-0454-1 PMC5602001 28989188

[36] Plano Clark VL. Meaningful integration within mixed methods studies: identifying why, what, when, and how. Contemp Educ Psychol. 2019;57:106–11.

[37] Pinnock H, Barwick M, Carpenter CR, Eldridge S, Grandes G, Griffiths CJ, et al. Standards for reporting implementation studies (StaRI) Statement. BMJ. 2017;356:i6795. 28264797 10.1136/bmj.i6795 PMC5421438

[38] Grol R, Wensing M, Eccles M, Davis D. Improving Patient Care: The Implementation of Change in Health Care, Second Edition. 2nd ed: John Wiley & Sons, Ltd; 2013.

[39] Proctor EK, Powell BJ, McMillen JC. Implementation strategies: recommendations for specifying and reporting. Implement Sci. 2013;8:139. 24289295 10.1186/1748-5908-8-139 PMC3882890

[40] Song MJ, Ward J, Choi F, Nikoo M, Frank A, Shams F, et al. A process evaluation of a web-based Mental Health Portal (WalkAlong) using Google Analytics. JMIR Ment Health. 2018;5(3):e50. 30126832 10.2196/mental.8594 PMC6121139

[41] Kessler RS, Purcell EP, Glasgow RE, Klesges LM, Benkeser RM, Peek CJ. What does it mean to employ the RE-AIM model? Eval Health Prof. 2013;36(1):44–66. 22615498 10.1177/0163278712446066

[42] Glasgow RE, Vogt TM, Boles SM. Evaluating the public health impact of health promotion interventions: the RE-AIM framework. Am J Public Health. 1999;89(9):1322–7. 10474547 10.2105/ajph.89.9.1322 PMC1508772

[43] Holtrop JS, Estabrooks PA, Gaglio B, Harden SM, Kessler RS, King DK, et al. Understanding and applying the RE-AIM framework: clarifications and resources. J Clin Transl Sci. 2021;5(1):e126. 34367671 10.1017/cts.2021.789 PMC8327549

[44] Kallio H, Pietilä AM, Johnson M, Kangasniemi M. Systematic methodological review: developing a framework for a qualitative semi-structured interview guide. J Adv Nurs. 2016;72(12):2954–65. 27221824 10.1111/jan.13031

[45] Archibald MM, Ambagtsheer RC, Casey CG, Lawless M. Using zoom videoconferencing for qualitative data Collection: perceptions and experiences of researchers and participants. Int J Qualitative Methods. 2019;18:1–8.

[46] Cadogan SL, McHugh SM, Bradley CP, Browne JP, Cahill MR. General practitioner views on the determinants of test ordering: a theory-based qualitative approach to the development of an intervention to improve immunoglobulin requests in primary care. Implement Sci. 2016;11(1):102. 27435839 10.1186/s13012-016-0465-8 PMC4952272

[47] QualRIS. Qualitative Methods in Implementation Science. 2019 https://cancercontrol.cancer.gov/IS/docs/NCI-DCCPS-ImplementationScience-WhitePaper.pdf. Accessed on 5 Sept 2024.

[48] Keyworth C, Hart J, Armitage CJ, Tully MP. What maximizes the effectiveness and implementation of technology-based interventions to support healthcare professional practice? A systematic literature review. BMC Med Inf Decis Mak. 2018;18(1):93. 10.1186/s12911-018-0661-3 PMC6223001 30404638

[49] Granja C, Janssen W, Johansen MA. Factors determining the success and failure of eHealth interventions: systematic review of the literature. J Med Internet Res. 2018;20(5):e10235. 29716883 10.2196/10235 PMC5954232

[50] Schreiweis B, Pobiruchin M, Strotbaum V, Suleder J, Wiesner M, Bergh B. Barriers and facilitators to the implementation of eHealth services: systematic literature analysis. J Med Internet Res. 2019;21(11):e14197–e. 31755869 10.2196/14197 PMC6898891

[51] Kruse C, Betancourt J, Ortiz S, Valdes Luna SM, Bamrah IK, Segovia N. Barriers to the Use of Mobile Health in improving Health outcomes in developing countries: systematic review. J Med Internet Res. 2019;21(10):e13263. 31593543 10.2196/13263 PMC6811771

[52] Wong SS, Lim HM, Chin AJZ, Chang FWS, Yip KC, Teo CH, et al. eHealth literacy of patients attending a primary care clinic in Malaysia and its associated factors: a cross-sectional study. Digit Health. 2022;8:1–10. 10.1177/20552076221135392 PMC9677303 36420318

[53] Krist AH, Phillips SM, Sabo RT, Balasubramanian BA, Heurtin-Roberts S, Ory MG, et al. Adoption, reach, implementation, and maintenance of a behavioral and mental health assessment in primary care. Ann Fam Med. 2014;12(6):525–33. 25384814 10.1370/afm.1710 PMC4226773

[54] Maskell K, McDonald P, Paudyal P. Effectiveness of health education materials in general practice waiting rooms: a cross-sectional study. Br J Gen Pract. 2018;68(677):e869–76. 30348885 10.3399/bjgp18X699773 PMC6255223

[55] Ward K, Hawthorne K. Do patients read health promotion posters in the waiting room? A study in one general practice. Br J Gen Pract. 1994;44(389):583–5. 7748670 PMC1239083

[56] Karia CT, Hughes A, Carr S. Uses of quick response codes in healthcare education: a scoping review. BMC Med Educ. 2019;19(1):456. 31810464 10.1186/s12909-019-1876-4 PMC6896690

[57] Sharara S, Radia S. Quick response (QR) codes for patient information delivery: a digital innovation during the coronavirus pandemic. J Orthod. 2022;49(1):89–97. 34308694 10.1177/14653125211031568

[58] Ross J, Stevenson F, Lau R, Murray E. Factors that influence the implementation of e-health: a systematic review of systematic reviews (an update). Implement Sci. 2016;11(1):146. 27782832 10.1186/s13012-016-0510-7 PMC5080780

[59] Santos WJ, Graham ID, Lalonde M, Demery Varin M, Squires JE. The effectiveness of champions in implementing innovations in health care: a systematic review. Implement Sci Commun. 2022;3(1):80. 35869516 10.1186/s43058-022-00315-0 PMC9308185

[60] Wood K, Giannopoulos V, Louie E, Baillie A, Uribe G, Lee KS, et al. The role of clinical champions in facilitating the use of evidence-based practice in drug and alcohol and mental health settings: a systematic review. Implement Res Pract. 2020;1:1–11. 10.1177/2633489520959072 PMC9924254 37089122

[61] Curran GM, Bauer M, Mittman B, Pyne JM, Stetler C. Effectiveness-implementation hybrid designs: combining elements of clinical effectiveness and implementation research to enhance public health impact. Med Care. 2012;50(3):217–26. 22310560 10.1097/MLR.0b013e3182408812 PMC3731143

