# Supplementary material for: Pilot implementation study of a web-based men’s health screening app in primary care during COVID-19: a mixed-methods approach
Source: BMC Health Serv Res. 2024 Oct 11;24:1219. doi: 10.1186/s12913-024-11702-9 (PMC11468301; doi:10.1186/s12913-024-11702-9)
Supplement: Supplementary file 1 — Supplementary Material 1. [file 12913_2024_11702_MOESM1_ESM.docx]

Additional file 1: Tailored implementation intervention for ScreenMen

| **Phase I: Engagement of key stakeholders** | |
| --- | --- |
| Name it | Strategy: Mandate change. |
| Define it | Have the clinic leader (Family Medicine Specialist) declare the priority of ScreenMen and their determination to have it implemented by coming out with an official memo. |
| Actor | Clinic leader. |
| Action | Issuing an official memo regarding the implementation of ScreenMen. |
| Action target | Healthcare providers comprised of doctors/nurses/medical assistants/registration counter healthcare providers. |
| Temporality | Before the implementation of the app. |
| Dose | Once during the implementation period. |
| Implementation outcome affected | Adoption of ScreenMen. |
| Justification | Issuing an official mandate showed that the clinic leader endorsed the implementation and this would motivate the healthcare providers to implement. |

| **Phase II: Strategies before implementation of ScreenMen** | | | |
| --- | --- | --- | --- |
| Name it | Strategy IIa: Provide education and training. | Strategy IIb: Identify and prepare champions. | Strategy IIc: The use of information and communication technology |
| Define it | - Conducting education and training workshops for healthcare providers. - Develop educational materials in the form of pamphlets/posters/buntings. | Identify a champion for ScreenMen. | - Use the official Facebook page of the clinic to promote the app. |
| Actor | The researchers. | Clinic leader and researchers. | The researchers. |
| Action | - Small group, train by batches. - Tailor training to specific groups. - Define specific roles & functions. - Help healthcare providers to see the value in screening. - Educate the importance of men’s health screening. - Educate about the action needed after patients completed ScreenMen. - Demonstrate how to use ScreenMen and have the participants use it during the workshop. - Produce patient pamphlets in three languages with QR codes to access the app. - Produce catchphrases in educational materials. | Appoint preferably a male healthcare provider to champion the implementation. | Promotion material to be posted on the Facebook page. |
| Action target | Healthcare providers comprised of doctors/nurses/medical assistants/registration counter healthcare providers. | Healthcare providers comprised of doctors/ nurses/medical assistants /registration counter healthcare providers. | Healthcare providers in charge of the clinic’s Facebook page. |
| Temporality | Before the implementation of the app. | Before the implementation of the app. | Before the implementation of the app. |
| Dose | One workshop for each batch of healthcare providers. | - | - |
| Implementation outcome affected | - Acceptability - Adoption - Appropriateness - Feasibility - Fidelity to the counselling of using ScreenMen. | - Acceptability - Adoption - Appropriateness - Feasibility - Penetration | - Feasibility - Penetration |
| Justification | Education and training to prepare the healthcare providers before the implementation is important to help them familiarise themselves with the app and how to implement it. | Having a champion to lead will help in the implementation. | Posting promotional materials on social media can be a way to promote ScreenMen to the community. |

| **Phase III: Strategy during the implementation of ScreenMen** | |
| --- | --- |
| Name it | Strategy IIIa: Audit and provide feedback. |
| Define it | Use WhatsApp as a platform to troubleshoot and improve the implementation of ScreenMen. |
| Actor | The researchers. |
| Action | - Set up a WhatsApp group with all healthcare providers involved in the implementation to troubleshoot and improve the implementation of the app. - Set up different groups for different categories of healthcare providers. |
| Action target | Healthcare providers comprised of doctors/nurses/medical assistants/registration counter healthcare providers. |
| Temporality | Throughout the implementation duration. |
| Dose | - |
| Implementation outcome affected | - Adoption - Penetration |
| Justification | Monitoring and feedback will be important to troubleshoot and resolve issues that might occur throughout the implementation process. |
