# Supplementary material for: Pilot implementation study of a web-based men’s health screening app in primary care during COVID-19: a mixed-methods approach
Source: BMC Health Serv Res. 2024 Oct 11;24:1219. doi: 10.1186/s12913-024-11702-9 (PMC11468301; doi:10.1186/s12913-024-11702-9)
Supplement: Supplementary file 2 — Supplementary Material 2. [file 12913_2024_11702_MOESM2_ESM.docx]

Additional file 2: Interview guide

| **RE-AIM component** | **Description** | **Interview Questions** |
| --- | --- | --- |
| Adoption | Factors affecting the participation of healthcare providers in adopting ScreenMen in daily practice.  Factors affecting the completion of ScreenMen by patients | 1. Can you please share your experience in promoting ScreenMen to the patient? 2. What motivated you to share ScreenMen with the patients? 3. What made it difficult to promote ScreenMen to the patients? 4. Any difficulty when promoting ScreenMen during the pandemic? 5. The completion rate for ScreenMen was low. Why do you think not many users completed the ScreenMen application? |
| Implementation | Factors affecting the implementation of ScreenMen daily practice. | 1. Which implementation strategy worked? Why? 2. Which implementation strategy did not work? Why not? 3. What other implementation strategies might have worked better? 4. ScreenMen was accessed using a few different modalities (Bunting, poster, Postcard, Facebook and direct). Bunting was the most used modality. What could be the reasons for this? Why did the other modalities not work as well as bunting?    1. What other modalities would be effective in promoting ScreenMen to the patients? |
| Maintenance | Intention to implement ScreenMen and factors influencing the decision. | 1. Would you like to continue using ScreenMen in KK Cheras Baru? 2. Would you recommend your colleagues (in KK CB, and other KK) to use it? If yes, why? If not, why not? 3. How can we expand the implementation of ScreenMen to a larger scale? |
| Reach | Factors affecting the participation of healthcare providers in the training workshop.  Factors influencing the usage of ScreenMen by patients. | 1. What do you think of the participation rate? Is this what you have expected? Why? 2. There was an increase in users starting in March 2021. What were the possible reasons for this? |
